# 

**Published:** 1894-08-25

**Authors:** 


					HOSPITAL. Aug. 25th, 1894.
DDI/Mr- xu/nnrMAr WW WITH EXTRA NURSING SUPPLEMENT.
.T WO PEN C E. . lEJIf ^ per Annum, 10s, 6d? post free.
,>-PR!CE TWOPENCE. ?sf^ M" ^
'^413- Vol. XVI.?Aug. 25th, 1894. HK Jrl jSf^
^fstered at the General Post Office as a Newspaper for
tranBniisnin-n in t.hn nnitfld Rinsrdom and Abroad.1 ^
Per Annnm, 10s. 6d., post free.
? ? V) ivii /?i i. '?Mb? ,wu ?? JMCy Monthly Parts, 6d. j post free, 8d.
istered at the General Post Office as a Newspaper for )W^ Ditto per Annum, 8s. 6d.,post free.
traii8mis8ioii in the United Kingdom and Abroad.] v/
/
/
I j
WHICH HOSPITAL
No- 413. Vol. XVI WITH EXTRA NURSING SUPPLEMENT. Saturday,
Aug. 25th, 1894.

				

